# Extracellular Matrix Rigidities Regulate the Tricarboxylic Acid Cycle and Antibiotic Resistance of Three‐Dimensionally Confined Bacterial Microcolonies

**DOI:** 10.1002/advs.202206153

**Published:** 2023-01-19

**Authors:** Yiming Han, Nan Jiang, Hongwei Xu, Zuoying Yuan, Jidong Xiu, Sheng Mao, Xiaozhi Liu, Jianyong Huang

**Affiliations:** ^1^ Department of Mechanics and Engineering Science, and Beijing Innovation Center for Engineering Science and Advanced Technology College of Engineering Peking University 100871 Beijing China; ^2^ Tianjin Key Laboratory of Epigenetics for Organ Development of Premature Infants Fifth Central Hospital of Tianjin Tianjin 300450 China

**Keywords:** bacterial biofilm, matrix stiffness, mechanobiology, metabolism, minimum biofilm elimination concentration

## Abstract

As a major cause of clinical chronic infection, microbial biofilms/microcolonies in host tissues essentially live in 3D‐constrained microenvironments, which potentially modulate their spatial self‐organization and morphodynamics. However, it still remains unclear whether and how mechanical cues of 3D confined microenvironments, for example, extracellular matrix (ECM) stiffness, exert an impact on antibiotic resistance of bacterial biofilms/microcolonies. With a high‐throughput antibiotic sensitivity testing (AST) platform, it is revealed that 3D ECM rigidities greatly modulate their resistance to diverse antibiotics. The microcolonies in 3D ECM with human tissue‐specific rigidities varying from 0.5 to 20 kPa show a ≈2–10 000‐fold increase in minimum inhibitory concentration, depending on the types of antibiotics. The authors subsequently identified that the increase in 3D ECM rigidities leads to the downregulation of the tricarboxylic acid (TCA) cycle, which is responsible for enhanced antibiotic resistance. Further, it is shown that fumarate, as a potentiator of TCA cycle activity, can alleviate the elevated antibiotic resistance and thus remarkably improve the efficacy of antibiotics against bacterial microcolonies in 3D confined ECM, as confirmed in the chronic infection mice model. These findings suggest fumarate can be employed as an antibiotic adjuvant to effectively treat infections induced by bacterial biofilms/microcolonies in a 3D‐confined environment.

## Graphical abstract

It remains elusive whether and how mechanical cures regulate antibiotic resistance of bacterial biofilms/microcolonies in 3D confined microenvironments. With a high‐throughput antibiotic sensitivity testing platform, this study not only reveals that 3D extracellular matrix rigidities enhance antibiotics resistance of microcolonies via the tricarboxylic acid cycle, but recognizes that fumarate can be used as an antibiotic adjuvant to treat infections.

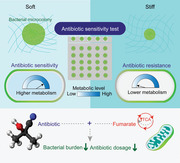

## 1 Introduction

Microorganisms that are ubiquitous on earth, often colonize as a community, that is, biofilms, both in the host and in the external environment.^[^ ^1^, ^2^ ^]^ The formation of bacterial biofilm in host tissues is a serious threat to cause chronic infections in patients.^[^ ^3^ ^]^ In fact, ≈80% of microbial infections have a biofilm‐related etiology.^[^ ^4^ ^]^ It has been demonstrated that biofilms can protect bacteria from immune attack and increase bacterial resistance to antibiotics,^[^ ^5^ ^]^ which is likely to lead to systemic inflammatory responses and surgical failure.^[^ ^6^ ^]^ Generally, in vivo bacterial biofilms in host tissues live in 3D constrained microenvironments, which exhibit some unique functions and behaviors essentially different in many respects from biofilms grown on two‐dimensional solid substrates, for example, changes in multidrug resistance (MDR), morphodynamics, genetic program, and metabolic activity.^[^ ^7^, ^8^, ^9^ ^]^ Particularly, it has been identified that many metabolic mutations, for example, central carbon and energy metabolism genes, can confer bacterial antimicrobial resistance (AMR) by increasing the minimum inhibitory concentrations (MICs) of various antibiotics by at least 60%.^[^ ^10^, ^11^, ^12^ ^]^ It is very difficult for conventional antibiotic sensitivity test (AST) methods based on 2D bacterial culture to precisely characterize the variation in drug resistance of bacterial biofilms living in 3D extracellular matrices, which likely gives a misleading MIC.^[^ ^4^, ^9^, ^13^ ^]^ For instance, there is evidence indicating that antimicrobial treatment determined by the traditional AST methods becomes ineffective in clinics once the biofilms have formed in patients with cystic fibrosis caused by *Pseudomonas aeruginosa* infection.^[^ ^14^ ^]^ Given the urgency of biofilm‐associated infections in the post‐antibiotic era, there is an urgent need to develop a more suitable in vitro AST model to determine the minimum biofilm elimination concentration (MBEC) in 3D confined ECM with specific physiological rigidities.^[^ ^15^, ^16^ ^]^

Essentially, the morphological evolution of bacterial biofilms/ microcolonies is directly related to ECM rigidities,^[^ ^7^ ^]^ which generally vary from 0.5 to 20 kPa in human tissues,^[^ ^17^ ^]^ depending on specific tissues and their pathophysiological states. In general, the higher the Young's modulus of ECM, the stronger its confinement ability to 3D bacterial biofilms/microcolonies.^[^ ^18^ ^]^ Recent progress implies that there exist remarkable changes in ECM stiffness during wound healing,^[^ ^19^ ^]^ pulmonary fibrosis,^[^ ^20^ ^]^ and progression of breast cancer.^[^ ^21^, ^22^ ^]^ These lesioned tissues often coexist with bacterial communities such that the bacterial biofilms in situ formed in these 3D lesions can further induce changes in local ECM rigidities and simultaneously exacerbate the aforementioned pathological processes.^[^ ^23^, ^24^ ^]^ On the other hand, the bacteria have already been shown to be mechanosensitive to the biomechanical microenvironments of ECM.^[^ ^25^ ^]^ Recent advances have revealed that the adhesion, proliferation, and antibiotic susceptivity of bacterial cells cultured on 2D flat solid substrates,^[^ ^26^, ^27^, ^28^ ^]^ and even their interactions with host cells^[^ ^29^ ^]^ are closely associated with mechanical rigidities of the 2D extracellular substrates. Likewise, multicellular organization, morphogenesis, and cell ordering in some constrained bacterial biofilms are modulated by 3D ECM stiffness.^[^ ^7^ ^]^ Normally, ECM stiffness can play a key role in regulating mechanobiological interactions between bacteria and ECM, and thus exert a critical influence on the secretion of extracellular polymeric substances (EPSs), which is crucial for the transition from 2D to 3D bacterial colonies.^[^ ^30^, ^31^, ^32^, ^33^ ^]^ Notably, the EPSs produced by the bacterial biofilms can also cause local heterogeneity in ECM architectures and allow for random changes in metabolism within isogenic populations. This may result in a fraction of phenotypically tolerant cells called persisters, which are largely responsible for the inability of antibiotics to completely eradicate infections.^[^ ^34^, ^35^ ^]^

Yet, whether and how ECM rigidities mediate antibiotic resistance of microbiofilms living in a 3D confined environment remains elusive. To this end, we first develop an antibiotic sensitivity testing (AST) assay based on photocrosslinkable methacrylated alginate (MA) hydrogels with tunable mechanical properties.^[^ ^36^, ^37^ ^]^ With the aid of a series of microwell arrays fabricated with the microfluidic chip‐based technique, the AST platform enables us to quantify the efficacy of antibiotics on bacterial microbiofilms grown in 3D confined ECM with various physiological rigidities in a high‐throughput fashion. Subsequently, we investigate spatiotemporal dynamics of bacterial microcolonies growth medicated by 3D ECM stiffness and explore the stiffness‐dependent resistance of bacterial microcolonies grown in 3D confined microenvironments to diverse antibiotics. Based on transcriptome sequencing and mechanobiological analyses, we reveal that 3D ECM rigidities play a crucial role in modulating the tricarboxylic acid (TCA) cycle of bacterial microcolonies living in 3D constrained microenvironments, which in turn dominates their antibiotic resistance. Finally, we show that fumarate, which is a potentiator of TCA cycle activity, can be utilized as an effective antibiotic adjuvant to alleviate the 3D ECM stiffness‐induced antibiotic resistance of biofilms, as confirmed in a mice‐based chronic infection model. These findings not only elucidate the inherent mechanobiological mechanism of 3D ECM stiffness promoting antibiotic resistance of bacterial microbiofilms but also present a feasible strategy to treat infections caused by biofilms/microcolonies in a 3D confined ECM with specific physiological rigidities.

## 2 Results

### 2.1 Spatiotemporal Dynamics of Bacterial Microcolony Growth in 3D Hydrogels with Different Rigidities

**Figure 1:**
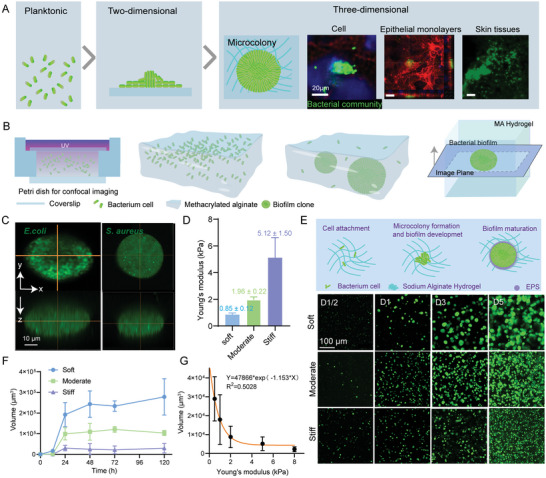
Formation of 3D bacterial microcolonies in MA hydrogels with tunable rigidities. A) Schematic of morphological features of bacterial microcolonies in a 2D or 3D environment. B) Schematic processes of forming bacterial microcolonies in 3D MA hydrogels. C) Representative 3D view (top and side) images for *E. coli* and *S. aureus* microcolonies embedded in a MA hydrogel block, where the bacterial cells constitutively expressed green fluorescent protein (GFP). Scale bar: 50 µm. D) Young's moduli of soft, moderate, and stiff MA hydrogels (*n* = 8). The data represented the mean ± SD from three independent experiments. E) Schematic of mature biofilm formation in the 3D hydrogels; bottom represented confocal images of *E. coli* microcolonies after 0.5, 1, 3, and 5 days of incubation in soft, moderate, and stiff MA hydrogels, respectively. F) Changes in volume of microcolonies after 0.5, 1, 3, and 5 days of incubation in soft moderate, and stiff hydrogels. At least *n* = 20 microbiofilms were analyzed at each time point. G) Quantitative dependence of the volume of microcolonies on Young's moduli of 3D confined hydrogels, where the bacteria embedded into the hydrogels was cultured for 24 h.

It is well‐known that the vast majority of bacteria tend to live in matrix‐embedded communities in their host in the form of aggregated microcolonies, usually termed 3D microbiofilms (Figure **1**A), rather than in a lifestyle of planktonic or 2D biofilms.^[^ ^10^ ^]^ Here, we adopted a biocompatible hydrogel material, that is, methacrylated alginate (MA), to mimic 3D ECM suitable for the growth and proliferation of bacterial biofilms. MA was synthesized through the well‐established standard carbodiimide chemistry.^[^ ^37^ ^]^ In the past decades, MA hydrogel had already been widely used as tissue engineering scaffold materials for 3D cell culture owing to its excellent biocompatibility and controllable mechanical properties.^[^ ^36^, ^37^, ^38^, ^39^ ^]^ In practice, the MA hydrogels were prepared by covalently photocrosslinking MA pregel solution with a certain amount of photoinitiator, that is, Irgacure 2959, which could quantitatively release free radicals when it was excited by 365 nm UV light. In this way, the MA hydrogels with different rigidities could be fabricated by controlling the amount of Irgacure 2959, UV light intensity, and curing time.^[^ ^40^ ^]^ To encapsulate single bacteria into the 3D MA hydrogel matrices, we first premixed bacterial suspension in the MA solution and then made it fully polymerize under 365 nm UV light to obtain the MA hydrogels with specific mechanical rigidities, which allowed the formation of bacterial biofilms in the 3D confined microenvironments (Figure 1B). One could readily observe anisotropic oblate biofilms of *Staphylococcus aureus* (ATCC 29213) and *Escherichia coli* (ATCC 25922) in the 3D MA hydrogels (Figure 1C). Also, it could be found that the bacteria tended to form biofilms with tightly aggregated states in the covalently crosslinked MA hydrogels, whereas they appeared to be loosely dispersed in the corresponding calcium ion‐crosslinked MA hydrogels (Figure S1A, Supporting Information). To mimic the lifestyle of the bacteria in 3D ECM and explore the regulatory mechanism of mechanical cues of ECM microenvironments, we subsequently fabricated the MA hydrogels with three different rigidities, hereafter referred to as soft (0.85 ± 0.12 kPa), moderate (1.96 ± 0.22 kPa), and stiff (5.12 ± 1.50 kPa), respectively (Figure 1D and Figure S1B, Supporting Information).

Originating from single‐cell planktonic bacteria, the formation of bacterial biofilms generally involves a complicated multistep process that includes at least three major steps of biofilm life, that is, aggregation, growth, and disaggregation independently of attached surfaces.^[^ ^41^ ^]^ Here, we investigated the spatiotemporal process of biofilm formation in 3D‐constrained situations by embedding single‐cell planktonic bacteria into the MA hydrogels with tunable mechanical rigidities. In all the soft, moderate, and stiff hydrogel samples, we could observe the formed microcolonies that were essentially different from those created on 2D flat solid surfaces. After individual planktonic bacteria were randomly embedded into the 3D hydrogels, they began to divide and clump together to in situ form microcolonies on days ≈1–3. After ≈3–5 days, these microbial films merged into larger communities and the corresponding EPS matrices were gradually deposited, eventually leading to the formation of mature biofilms (Figure 1E). The current in vitro culture model could effectively capture the full lifestyle and complex spatial microarchitectural development of bacterial biofilms grown in 3D confined ECM environments. The greater the matrix stiffness, the smaller the average size of the microcolonies (Figure 1F), which implied that their growth was greatly affected by the mechanical rigidities of surrounding hydrogels. Figure 1F quantitatively presented the time‐related average sizes of bacterial microcolonies grown in the 3D MA hydrogels with different rigidities. Within the initial 12 h of implantation of single‐cell planktonic bacteria into the hydrogel samples, there was no significant difference in the average volume of the microcolonies, irrespective of the mechanical stiffness of the hydrogels involved. However, 24 h after the bacteria were embedded into the hydrogel samples with different rigidities, one could detect significant volume differences of the bacterial microcolonies (Figure 1G), suggesting that 3D ECM stiffness‐dependent mechanical confinement effect gradually emerged during the formation of bacterial microcolonies. Additionally, it turned out that the permeability (Figure S1C, Supporting Information) and degradability (Figure S1D,E, Supporting Information) of the MA hydrogels exerted little influence on the growth of bacterial microcolonies. In general, biofilms displayed viscoelastic properties, depending strongly on the forces acting on the EPS matrix.^[^ ^42^ ^]^ With the help of a well‐developed micropipette aspiration‐based experiment (Figure S2A,B, Supporting Information), we further characterized the aggregate viscosity of the microcolonies cultured in 3D confined microenvironments. It appeared that the viscosity took on an increasing trend with the increase in stiffness of the MA hydrogels. Quantitatively, they were 0.032 and 0.24 Pa s^−1^ for the bacterial microcolonies in the soft and stiff hydrogels, respectively (Figure S2C,D, Supporting Information). It suggested the higher stability of microcolonies in stiff hydrogels. Biofilms could increase the strength of their structural matrix in response to mechanical stresses by increasing EPS production.^[^ ^43^ ^]^ In addition, a previous study demonstrated that, in growing uropathogenic *E. coli* colonies, biofilm matrix deposition could be particularly high in areas of increased mechanical stresses in confined spaces.^[^ ^30^ ^]^ Thus, bacterial microcolonies that underwent a higher extent of geometric stress constraints in a relatively stiff matrix would produce more EPS. In nature, EPSs could improve the structural and chemical stability of bacterial biofilms^[^ ^44^, ^45^ ^]^ and therefore enhance their tolerance to antimicrobial agents.^[^ ^46^, ^47^ ^]^

### 2.2 High‐Throughput In Vitro 3D AST Assay Revealing Enhanced Antibiotic Resistance of Microcolonies Induced by 3D ECM Stiffness

**Figure 2:**
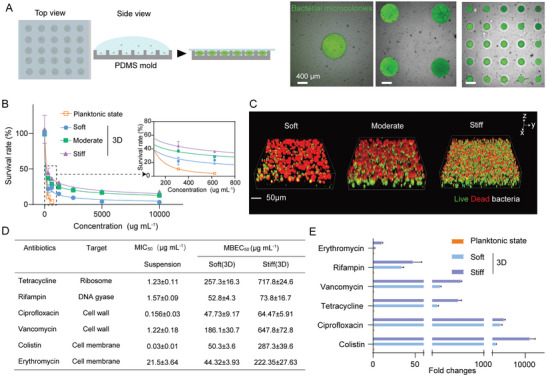
Mechanical rigidities promoted antibiotical resistance of bacterial microcolonies in the developed in vitro 3D AST model. A) Schematic diagram of a PDMS microwell array for in vitro 3D AST. The three sub‐images on the right showed representative ones of bacterial microcolonies cultured in the PDMS microwell arrays whose depths were 300 µm while the corresponding diameters were 200, 800, and 1000 µm, respectively. Note that the bacterial microcolonies were beforehand labeled as green in experiments. Scale bar: 400 µm. B) Quantitative dependence of survival rates of planktonic *E. coli*, the corresponding microcolonies grown in soft, moderate, and stiff MA hydrogel samples on concentrations of broth microdilution erythromycin (0‐10 000 µg mL^−1^). C) Results of live/dead staining for *E. coli* microcolonies in soft, moderate, and stiff MA hydrogels, respectively, after treatments with 5000 µg mL^−1^ erythromycin. The live and dead bacterial cells were labeled green and red, respectively. Scale bar: 50 µm. D) MICs and MBECs of planktonic *E. coli* and the corresponding microcolonies cultured in soft and stiff MA hydrogels after treatments with various antibiotics with distinct targets. Data were presented as mean±SD from at least three individual experiments. E) Ratios of MBECs of bacterial biofilms grown in soft and stiff MA hydrogels to MIC of the corresponding planktonic bacteria after they were treated with various antibiotics.

Based on the well‐developed soft lithography and microfluidic technique, we fabricated a series of PDMS microwell arrays in order to realize high‐throughput in vitro 3D AST (**Figure** **2**A). The depths of the fabricated microwells were designed as 300 µm, whereas their diameters were 200, 800, and 1000 µm, respectively. The microwells were filled with MA pregel solutions mixed with a certain amount of single‐cell planktonic *E. coli*, followed by photocrosslinking with UV light of 365 nm. Afterward, they were incubated at 37 °C for 48 h to form mature bacterial microcolonies in the 3D confined microenvironments (Figure 2A). We then added various antibiotic solutions with a particular gradient concentration varying from ≈0–10 000 µg mL^−1^ to treat the bacterial microcolonies for at least 24 h. The broth microdilution method was employed to quantify the minimum inhibition concentrations (MICs) of six classes of antibiotics for the bacteria in the planktonic state, while the minimum biofilm eradicating concentrations (MBECs) of the antibiotics for the corresponding bacterial microcolonies in the 3D MA hydrogels were determined via measuring the absorbance of the culture broth and live/dead staining in the current experiments. Figure 2B displayed the results of erythromycin‐sensitive tests for planktonic *E. coli* and the corresponding microcolonies grown in soft, moderate, and stiff MA hydrogels, where the adopted concentration of broth microdilution erythromycin ranged from ≈0–10 000 µg mL^−1^. One could observe the L‐shaped curves of survival rates of the planktonic *E. coli* and the related microcolonies versus erythromycin concentration (Figure 2B). It turned out that 21.5 µg mL^−1^ of erythromycin could inhibit the growth/proliferation of 50% *E. coli* growth under the planktonic condition. By contrast, it required more than 10 000 µg mL^−1^ of erythromycin to effectively prevent the growth/proliferation of the *E. coli* microcolonies in the 3D constrained situations (Figure 2B), which was consistent with previous reports that MBECs for bacterial biofilms in 3D culture cases were higher than MICs for the corresponding planktonic bacteria.^[^ ^3^, ^4^ ^]^ Interestingly, we further found that MBECs for the microcolonies in 3D confined situations were essentially a function of 3D ECM/hydrogel rigidities. For example, Figure 2B revealed that MBEC for *E. coli* microcolonies grown in the stiff hydrogels (222.35 µg mL^−1^) was much higher than that for the microcolonies cultured in soft ones (44.32 µg mL^−1^), indicating that MBEC had a significant upward trend with increasing 3D ECM stiffness. At the same time, the percentage of dead bacteria in the bacterial microcolonies grown in the stiff hydrogels was also much lower than that in the soft hydrogels when they were treated with 10 000 µg mL^−1^ of erythromycin for 24 h (Figure 2C). Specifically, the microcolonies in 3D ECM with human tissue‐specific rigidities displayed a ≈2–10 000‐fold increase in MBEC/MIC, depending on the types of antibiotics (Figures 2D,E).

Further, we tried treating the bacterial microcolonies in 3D hydrogels with specific mechanical rigidities with other antibiotics with distinct targets including ciprofloxacin, rifampin, tetracycline, colistin, and vancomycin, respectively. A similar phenomenon could be detected for these five antibiotics with different targeting mechanisms (Figure 2D), which implied that the 3D stiffness‐dependent enhancement in antibiotical resistance of bacterial microcolonies was likely to be universal. This point indicated that the extent of 3D mechanical confinement for bacterial microcolonies modulated ECM/hydrogel rigidities might trigger a cascade of mechanotransduction pathways to regulate their antibiotical resistance. A systematic comparative analysis demonstrated that all those MBECs for the microcolonies cultured in the stiff hydrogels were ≈1–5 times higher than those in the soft hydrogels (Figure 2E and Figure S3, Supporting Information). Among all the treated groups, it seemed that bacterial microcolony resistance to vancomycin and colistin was more sensitive to changes in the 3D mechanical stiffness of the MA hydrogels adopted in the current experiments (Figure 2E and Figure S3, Supporting Information). A possible reason was that the confined ECM/hydrogels might promote EPS‐related structural and chemical stability of bacterial microcolonies, which made it more difficult for these two antibiotics to disrupt bacterial cell walls and membranes in the microcolonies and thus enhanced their tolerance to the antibiotics.

### 2.3 ECM stiffness Regulating Transcriptional Profiles of Bacterial Microcolonies grown in 3D Confined Microenvironments

**Figure 3:**
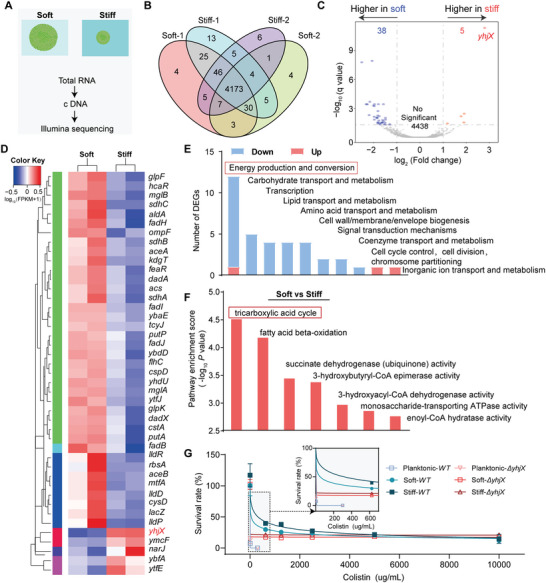
Bacterial microcolonies living in soft and stiff hydrogels showed distinct transcriptional profiles. A) Experimental procedures for transcriptional sequencing of bacterial microcolonies grown in soft and stiff hydrogels. B) Venn diagram of transcriptomes bacterial microcolonies in the soft and stiff hydrogels. C) Volcano map of differentially expressed genes (DEGs) for the bacterial microcolonies grown in soft and stiff hydrogels, which were plotted in logarithmic coordinates. D) Cluster map of differential genes expressed in the bacterial microcolonies grown in the soft and stiff hydrogels, where red represented highly expressed genes while blue denoted low expressed ones. E) Distribution map of clusters of orthologous groups (COGs), in which the horizontal axis represented the COG classification while the vertical axis presented the number of differential genes. Blue and red bars denoted down‐regulated and up‐regulated genes, respectively. F) Gene ontology (GO) enrichment analysis (*p*‐value < 0.05), where the horizontal axis listed the enrichment GO terms whereas the vertical axis showed their respective values of ‐log_10_ (*p*‐value), which quantified the extent of GO enrichment of the enrichment GO terms regulated by 3D hydrogel stiffness. The higher the value, the more significant the degree of enrichment. G) Quantitative dependence of survival rates of *WT* and *ΔyhjX E. coli* ATCC 25922. The bacteria were cultured in the planktonic state, soft, and stiff MA hydrogel samples with the concentration of broth microdilution colistin ranging from 0 to 10 000 µg mL^−1^. Data were presented as mean ± SD from at least three individual experiments (*n* = 8).

To dissect the inherent mechanobiological mechanism that ECM/hydrogel stiffness affected antibiotic resistance of bacterial microcolonies in 3D confined microsurroundings, we employed a transcriptomic approach to sequence the transcriptional profiles of the microcolonies grown in soft and stiff hydrogels, respectively (**Figure** **3**A). A significant number of differentially expressed genes (DEGs) were detected when comparing the gene sequencing results of the microcolonies grown in soft and stiff hydrogels (Figure 3B), which suggested that 3D ECM/hydrogel rigidities played a nonnegligible role in mediating specific gene expression. Further, we found that five DEGs (*yhjX*, *ymcF*, *narJ*, *ybfA*, and *ytfE*) were up‐regulated whereas thirty‐eight ones were down‐regulated in the bacterial microcolonies cultured in the 3D hydrogels when the hydrogel stiffness increased (Figure 3C,D). It could be found that, in the up‐regulated genes, *yhjX* had the highest expression level for the bacterial microcolonies grown in the stiff hydrogels, implying that it was most sensitive to fluctuations in 3D ECM/hydrogel stiffness (Figure 3C). The gene *yhjX* encoded a putative major facilitator superfamily (MFS) type transporter with 12 predicted transmembrane helices. As one of the two largest families of membrane transporters, MFS was ubiquitous in bacteria, which could transport a wide range of compounds, like sugar, oligosaccharides, drugs, amino acids, nucleosides, metabolites, and a large number of anions and cations. Also, *yhjX* was known to limit bacterial growth under specific stress conditions by controlling nutrient consumption.^[^ ^48^ ^–^ ^51^ ^]^ Additionally, *yhjX* was recognized to modulate the growth of *E. coli* in the presence of a subinhibitory concentration of gentamicin and simultaneously mediate the adaptive resistance to gentamicin.^[^ ^49^ ^]^ Taken together, it was speculated that *yhjX* was one of the key downstream genes associated with the mechanosensing of bacterial microcolonies cultured in 3D‐constrained microenvironments. Thus, we investigated the effect of the gene *yhjX* on antibiotic resistance of planktonic bacteria and bacterial microcolonies grown in the 3D matrix. The sensitivity of *ΔyhjX E. coli* to colistin did not alter significantly in the planktonic state. However, microcolonies in 3D matrices exhibited significantly increased sensitivity to colistin. Notably, *ΔyhjX* microcolonies cultured in soft and stiff matrices showed similar antibiotic sensitivity (Figure 3G). This suggested that the up‐regulated *yhjX* contributed to increased antibiotic resistance of microcolonies in the stiff hydrogel.

Likewise, most of the down‐regulated DEGs were rich in the pathways related to energy production and conversion (Figure 3E). Specifically, the pathways concerning the tricarboxylic acid (TCA) cycle exhibited the highest enrichment score in the bacterial microcolonies grown in the stiff hydrogel samples (Figure 3F). It demonstrated that the expression of *yhjX* was highly and specifically induced by pyruvate in various *E. coli* strains.^[^ ^49^ ^]^ In essence, pyruvate could form acetyl coenzyme A under the action of pyruvate dehydrogenase in the inner mitochondrial membrane. Acetyl coenzyme A was not only an important intermediate metabolite in the metabolism of energy substances but also a pivotal substance in the core metabolism of cells.^[^ ^52^ ^]^ Thus, the up‐regulated expression of *yhjX* accounted for a drastic decrease in core metabolic levels within the bacterial colonies (Figure 3E,F). These facts indicated that 3D ECM stiffness‐triggered mechanosensing and mechanotransduction pathways could exert a crucial effect on the TCA cycle and therefore alter the central carbon and energy metabolism in the microcolonies cultured in 3D confined microenvironments, which was in turn implicated in their antibiotic resistance, as confirmed in some previous literature^[^ ^10^, ^11^, ^53^ ^]^ which showed that inactivation of the TCA cycle could enhance the persister cell formation in the stationary phase.

### 2.4 Metabolism‐Dependent Antibiotic Resistance of Microcolonies Regulated by 3D ECM Rigidities

**Figure 4:**
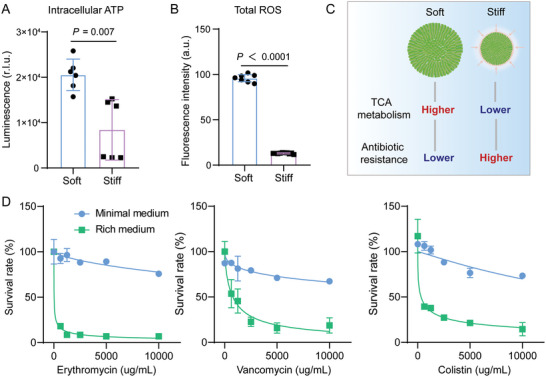
Increased metabolic levels promoted the clearance of bacterial microcolonies. A) Intracellular ATP and B) total reactive oxygen species (ROS) of bacterial microcolonies grown in soft and stiff hydrogels. Results were displayed as mean ± SD (*n* = 6). C) Schematic diagram describing that an increase in 3D ECM/hydrogel rigidities inhibited TCA metabolism, which in turn promoted antibiotic resistance of bacterial microcolonies cultured in 3D confined microenvironments. D) Dependence of survival rates of bacterial microcolonies in 3D MA hydrogels on the rich medium and the minimal medium (0.04% glucose) containing erythromycin, vancomycin, and colistin, respectively. The concentrations of these three antibiotics range from 0 to 10 000 µg mL^−1^. Prior to quantification of the survival rates, these antibiotics were employed to treat the microcolonies in 3D confined situations for 12 h, respectively. Data were presented as mean ± SD from three different experiments.

The TCA cycle was essential for bacterial uptake of energy and nutrients, which generally produced considerable amounts of adenosine triphosphate (ATP) molecules and reactive oxygen species (ROS) accumulation in bacterial biofilms and thereby exerted a crucial impact on the lethality of antibiotic treatments.^[^ ^54^ ^]^ In the current experiments, it could be found that the amount of intracellular ATP molecules produced by bacterial microcolonies grown in stiff hydrogels was much lower than those in soft hydrogels (**Figure** **4**A). Also, the ROS accumulation for bacterial microcolonies cultured in stiff hydrogels was fivefold lower than that for microcolonies in soft hydrogels (Figure 4B), which implied that an increase in 3D ECM/hydrogel rigidities was able to significantly inhibit the intracellular ROS accumulation. These indicated that there was a lower level of TCA metabolism in the bacterial microcolonies grown in stiff hydrogels, which might present higher antibiotic resistance, as illustrated in Figure 4C. To further determine the regulatory relationship between metabolic levels of the bacterial microcolonies and the corresponding antibiotic resistance, we subsequently treated the mature microcolonies grown in the stiff hydrogels with three representative antibiotics, that is, erythromycin, vancomycin or colistin, with different concentrations in the MOPS EZ rich defined medium (hereafter referred to as “rich medium”) or MOPS minimal media supplemented with 0.04% glucose (hereafter referred to as “minimal medium”), respectively.

Figure 4D presented the experimental curves of survival rates of microcolonies in the stiff hydrogels versus concentrations of the specific antibiotics. It indicated that the survival rates of microcolonies in the 3D‐constrained hydrogels were significantly reduced when they were treated with the rich medium containing erythromycin, vancomycin, or colistin. In comparison, they were less affected by the minimal medium with the same antibiotical concentration (Figure 4D). In particular, the lethality of antibiotics to the microcolonies could arrive at ≈60% to 80% when treated with the antibiotics of 10 000 µg mL^−1^ in the rich medium, depending on the type of the specific antibiotics, while it was less than 50% after the microcolonies were treated with the minimal medium containing the same concentration of antibiotics. Considering that the rich medium could promote the metabolism of bacterial biofilms whereas the minimal medium might inhibit their metabolic levels,^[^ ^12^ ^]^ one could confirm that an increase in the metabolic level in the bacterial microcolonies could effectively enhance the lethality of antibiotics, thereby facilitating the clearance of microcolonies grown in 3D confined microenvironments.

### 2.5 Fumarate as an Adjuvant Capable of Significantly Enhancing Therapeutic Efficacy of Antibiotics Against Bacterial Microcolonies grown in 3D Confined Microenvironments

**Figure 5:**
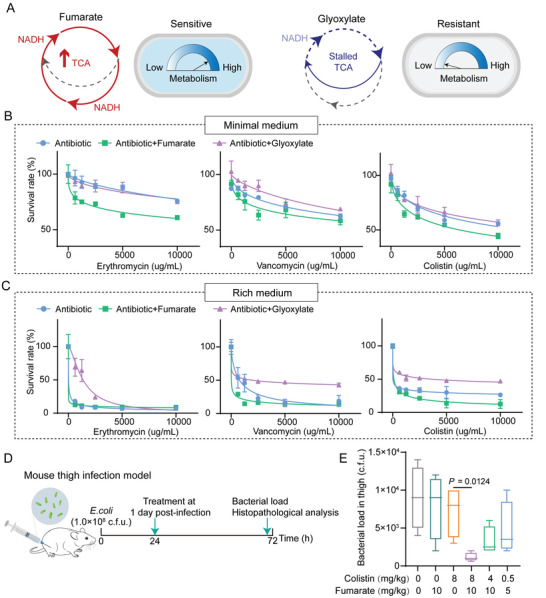
Fumarate and glyoxylate regulated antibiotic sensitivity of bacterial microcolonies grown in 3D confined microenvironments. A) Schematic diagrams illustrated that fumarate accelerated the TCA cycle whereas glyoxylate stalled the metabolic process, which could therefore modulate antibiotic resistance of bacterial cells/microcolonies. Notice that NADH (Nicotinamide adenine dinucleotide) was a necessary redox factor that transferred electrons from the TCA cycle to create energy in the ATP form. B) Curves of survival rates of mature bacterial microcolonies in 3D MA hydrogels regulated by fumarate and glyoxylate. In these experiments, the bacterial microcolonies were treated for 4 h with the minimal medium (0.04% glucose) containing a specific antibiotic (i.e., erythromycin, vancomycin, or colistin) in the presence of fumarate (15 mM) or glyoxylate (30 mM). All data were shown as mean ± SD (*n* = 6). C) The descriptions for the curves were the same as those in (B) except that the minimal medium was replaced with the rich medium in the experiments. D) Schematic illustration of experimental protocols for the adopted mouse thigh infection model. E) Statistical results of bacterial load in the mouse thigh infection model (*n* =  6). The bacterial load of the right thigh muscles infected with a non‐lethal dose of *E. coli* (1.0 × 10^8^ CFU) showed a dramatically decreasing trend after a single intraperitoneal injection of colistin (8 mg kg^−1^) plus fumarate (10 mg kg^−1^). *p*‐values were determined using a two‐sided, Mann–Whitney U‐test. All data were presented as mean ± SD.

We quantitatively evaluated the effect of TCA metabolite stimulations on antibiotics sensitivity of mature *E. coli* microcolonies cultured in 3D stiff hydrogels. The components of the lower part of the TCA cycle (e.g., fumarate and succinate) were utilized to potentiate the TCA cycle activity, thereby increasing downstream cellular respiration and proton motive force,^[^ ^55^ ^]^ while the upper TCA cycle metabolites (e.g., citrate and glyoxylate) were introduced to divert carbon flux away from the TCA cycle and collapse cellular respiration (**Figure** **5**A and Figure S5A–C, Supporting Information).^[^ ^10^, ^56^ ^]^ We treated bacterial microcolonies in the stiff matrix with gradient dilutions of colistin (0–10 000 µg mL^−1^) supplemented with each carbon source normalized to deliver 60 mM total carbon in the minimal medium. Interestingly, the components of the lower part of the TCA cycle (i.e., fumarate and succinate) were quite effective in enhancing the susceptibility of bacterial colonies to antibiotics, whereas the upper TCA cycle metabolites (i.e., citrate and glyoxylate) appeared to have little effect (Figure 5B,C and Figure S5D, Supporting Information). Specifically, when fumarate or succinate was combined with colistin, the efficacy of colistin against 3D bacterial microcolonies increased by 2‐fold. In contrast, the removal efficiency of colistin for the bacterial microcolonies was significantly reduced after the introduction of glyoxalate (Figure S5E, Supporting Information). Furthermore, we selected fumarate and glyoxylate as typical metabolites to investigate their effect on bacterial microcolonies in the minimal and rich medium containing serially diluted erythromycin, vancomycin, and colistin (≈0–10 000 µg mL^−1^). Our experimental data showed that glyoxylate could significantly protect the bacterial microcolonies, but fumarate increased antibiotic sensitivity of the bacterial microcolonies in both the minimal and rich media, (Figure 5B,C). These findings demonstrated that inhibition of the TCA metabolism could effectively improve antibiotic resistance of microcolonies grown in 3D confined microenvironments. Conversely, the enhanced TCA metabolism could increase antibiotic susceptibility of bacterial microcolonies in 3D hydrogels (Figure 5B,C).

**Figure 6:**
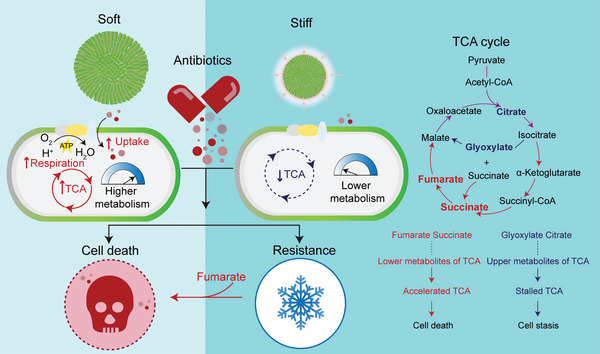
ECM rigidities modulated the TCA cycle and antibiotic resistance of 3D confined bacterial microcolonies.

We further assessed the therapeutic effect associated with fumarate based on a mouse thigh infection model (Figure 5D), which was reported as an ideal model for the studies on bacterial biofilm‐associated chronic abscess infections.^[^ ^57^, ^58^ ^]^ Experimental results from the animal model displayed a significant decrease in the bacterial burden in the right thigh muscles (Figure 5E) after a single intraperitoneal injection of colistin (8 mg kg^−1^) plus fumarate (10 mg kg^−1^), which was further confirmed by the alleviated pathological changes in thigh musculature of infected sites (Figure S6A, Supporting Information). Besides, none of the therapeutic doses adopted in the investigation showed any significant visceral toxicity (Figure S6B, Supporting Information). These findings indicated that fumarate could be used as an antibiotic adjuvant to promote the lethality of antibiotics and more effectively treat the infections induced by bacterial microcolonies in 3D confined microenvironments. **Figure** **6** further summarized the mechanism by which ECM rigidities mediated the TCA cycle and antibiotic resistance of 3D confined bacterial microcolonies.

## 3 Discussion and Conclusion

The vast majority of persistent infections in humans originate from bacterial biofilms.^[^ ^5^ ^]^ The infection triggered by the biofilms/microcolonies in the host is usually accompanied by persistent low‐grade inflammation and probably develops into a chronic state. In the clinical setting, chronic infections caused by biofilms are normally classified as surface‐associated and non‐surface‐associated infections. The former is common in patients with implants or medical devices, whereas the latter includes respiratory tract infection or persistent soft tissue infection that is related to comorbidities such as diabetes and impaired vascularization of the lower limbs predisposing to non‐healing wounds. Chronic infectious diseases induced by bacterial biofilms are commonly refractory to antibiotic treatments.^[^ ^59^ ^]^ One of the main reasons behind this is that the biofilm‐dwelling cells have a great advantage over the solitary bacterial cells, including enhanced antibiotic tolerance.^[^ ^1^ ^]^ Nevertheless, the potential influence of mechanical aspects of 3D ECM microsurroundings, for example, ECM stiffness, on the embedded bacterial biofilms/microcolonies remains largely unknown, although most bacterial biofilms/microcolonies in vivo essentially live in 3D mechanically constrained microenvironments.

For this purpose, we presented a high‐throughput AST assay to investigate the mechanobiological mechanism that ECM rigidities modulated the functions and behaviors of bacterial microcolonies grown in 3D mechanically confined microenvironments. The high‐throughput AST assay differed from the conventional 2D methods because it fully considered the regulatory effects of ECM dimensions and mechanical constraints on the microcolonies. With the help of transcriptome sequencing, our experimental data uncovered that ECM rigidities not only played a key role in mediating spatiotemporal dynamics of microcolony growth in 3D mechanically constrained states but also largely dominated the TCA metabolic process via bacterial mechanotransduction and ultimately exerted a leading effect on their antibiotic resistance. As a matter of fact, at least three core mechanisms of antimicrobial tolerance have already been identified over the past decades, including impaired penetration of antibiotic molecules, metabolic heterogeneity, and activation of adaptive responses to stress and antibiotics.^[^ ^8^, ^15^ ^]^ The current mechanobiological investigations shed light on the intrinsic relationship between mechanical cues of ECM and the TCA metabolism of bacterial biofilms, thus providing a novel perspective for the development of alternative in vivo anti‐infective treatment strategies as well as studies on antibiotic resistance of bacterial biofilms in complicated biomechanical microsurroundings.

Previous investigations had shown that improved metabolic stimulation could kill both Gram‐negative (*E. coli*) and Gram‐positive (*S. aureus*) persistent bacteria in the planktonic state with aminoglycosides.^[^ ^60^ ^]^ Our experimental results further demonstrated that an increase in the metabolic level also contributed to the efficient clearance of bacterial biofilms grown in 3D confined situations. Inspired by these findings, we also identified some metabolic regulators like fumarate, that could be used as an antibiotic adjuvant to alleviate the stiffness‐regulated antibiotic resistance of biofilms/microcolonies grown in 3D confined microenvironments, as validated in the chronic infection mice model. It should be pointed out that fumarate was an intermediate in the TCA metabolic cycle with immunomodulatory properties, which had already been approved by the US Food and Drug Administration (FDA) for treating asthma.^[^ ^10^, ^61^ ^]^ Their derivatives such as dimethyl fumarate (DMF) were also candidates for the treatment of virus‐induced hyperinflammation.^[^ ^62^, ^63^ ^]^ Additionally, it was reported that co‐administration of fumarate with tobramycin might increase the success rate of bacterial eradication and significantly improve lung function in cystic fibrosis patients.^[^ ^64^ ^]^ These suggested that fumarate had excellent biosafety for patients, making it a promising antibiotic adjuvant to treat in vivo infections caused by bacterial microcolonies.

Taken together, the current work not only clarified the mechanobiological mechanism by which 3D ECM rigidity regulated antibiotic resistance in mature microcolonies but also paved the way for the clinical development of metabolic modulator‐based antibiotic treatments. Further work should improve current experimental platforms to mimic as closely as possible natural bacterial biofilms/ microcolonies grown in 3D confined microenvironments, which should possess ultra‐complex compositional, structural, and genetic heterogeneity.

## 4 Experimental Section

### Bacterial Strains

Bacterial strains used in this study included *E. coli* (ATCC 25922) and *S. aureus* (ATCC 29213), which had been beforehand transfected with the plasmid pSC19‐GFP (erythromycin resistance). Prior to experiments, the bacterial strains were routinely maintained on LB agar plates and stored frozen in glycerol (60% *v/v*) at −80 °C. In experiments, the bacterial strains were cultured in Luria–Bertani (LB, Land Bridge Technology) broth at 37 °C with shaking.

### Preparation of Methacrylate Alginate (MA) Hydrogels

Alginate (molecular weight: ≈250 kDa, high G blocks; Novamatrix UP MVG) was beforehand oxidized with sodium periodate (1.5%) for ≈12 h at room temperature. Then the reaction was quenched by dropwise addition of ethylene glycol for 45 min. Further, The solution was dialyzed against deionized (DI) water for 3 days and then lyophilized. For 3D cell culture, the MA product was dissolved in Dulbecco's phosphate‐buffered saline (DPBS) solution (2%, w/v) and shaken at the speed of 120 rpm. for ≈24 h to ensure that it was fully dissolved. Subsequently, the MA solution was stored at 4 °C until use. The MA hydrogels with different rigidities were prepared by adjusting the amount of photoinitiator (Irgacure 2959, Sigma‐Aldrich) and the curing time, as summarized in Table S1, Supporting Information.

### 3D Culture of Bacterial Microcolonies

Prior to encapsulation in the MA hydrogels with specific rigidities, the bacterial cells were cultured at 37 °C with shaking at 200 rpm overnight. Subsequently, the suspension was washed and resuspended in the phosphate‐buffered saline (PBS, 0.01 mol L^−1^, pH 7.4) solution. The suspension of *E. coli* was then diluted at the density of 1 × 10^4^ CFU mL^−1^ for subsequent use. To achieve a 3D culture of bacterial cells in the MA hydrogels, 10 µL of the bacterial suspension was added into 100 µL of the pregel solution, mixed thoroughly on a vortexer to evenly disperse the bacteria, and cured them with UV light of 365 nm, as summarized in Table S1, Supporting Information. After the bacteria‐embedding hydrogels were cultured in the MOPS EZ‐rich media (Teknova, #M2105) at 37 °C for 24 h, the bacterial microcolonies were imaged with a Nikon A1 confocal microscope.

### Characterization of Mechanical Properties Based on Atomic Force Microscopy

The Young's moduli of the prepared MA hydrogels were probed by an atomic force microscope (AFM, MFP‐3D BIO, Asylum Research). A polystyrene microsphere (15 µm in diameter, Polysciences, Inc.) was first immobilized on an AFM probe (NP‐S type D nominal spring constant of 0.08 N m^−1^; Bruker Corporation) using a two‐component polyurethane glue (Bison International). The hydrogel samples immersed in 1 × PBS were quantified based on a standard protocol as described in Ref. [37].

### Micropipette Aspiration‐Based Experiments

Micropipette aspiration was carried out based on the protocol as previously described.^[^ ^65^ ^]^ In brief, pipettes were fabricated by pulling borosilicate capillaries (1/0.78 mm O/I diameter, Harvard Apparatus) with a laser‐based puller (P‐2000, Sutter Instruments). In practice, the micropipettes were sized to the desired diameter and then fire‐polished by using a microforge with a heated glass ball to generate a smooth glass surface. Prior to the micropipette aspiration, the MA hydrogels with mature bacterial microcolonies were lysed with 100 µL of alginate lyase (1 U mL^−1^) for 15 min at room temperature, and then the lysis reaction was terminated by adding 200 µL of DI water. Next, the liquid suspension of bacterial aggregates was transferred to a glass slide to facilitate the access of the micropipette. With the help of negative pressure of 100 Pa, this micropipette was able to partially aspirate bacterial microcolonies. The dynamic process concerning mechanical interactions between the micropipette and the microcolonies was recorded with a Nikon A1 confocal microscope at a rate of 1 frame s^−1^. all these images were analyzed with the Image J software.

### Fabrication of Microwell Array Chips

The PDMS microwell arrays were fabricated based on the well‐established soft lithography and microfluidic technique. Briefly, a silicon wafer was spin‐coated with SU8‐2050 negative photoresist at 1500 rpm. for 30 s to generate a layer of photoresist of 50 µm in thickness, which was then baked at 95 °C for 15 min. Next, the wafer with photoresist was exposed to UV light under a fabricated high‐resolution photomask with the designed microwell arrays based on a URE‐2000/35 ultraviolet lithography machine (Institute of Optics and Electronics, Chinese Academy of Sciences, China) for 75 s, and was further baked at 95 °C for 5 min.

Afterward, it was immersed in a developer (SUN‐238D, Suntific Materials Inc., China) for 60 s to remove the unexposed portions of the photoresist. Finally, the wafer with the microstructures was treated with hexamethyldisilazane (HDMS) under vacuum for 1 h to form hydrophobic surfaces. To fabricate PDMS microwell arrays, a premixed solution of PDMS base monomer and curing agent (10:1, w/w) was spin‐coated on silicon wafers and cured overnight at 65 °C. After the microwell arrays were plasm‐treated to enhance their hydrophilicity, the MA pre‐gel solution with single bacterial cells was loaded into the microwell array chips and then polymerized in situ with UV light of 365 nm in order to achieve a 3D culture of bacterial microcolonies in the MA hydrogels with specific stiffness.

### Antibiotic Susceptibility Testing (AST)

The MICs of antibiotics against planktonic bacteria were determined by the broth microdilution method according to the Clinical and Laboratory Standards Institute (CLSI) 2018 guidelines.^[^ ^66^ ^]^ The antibiotic stock solutions were diluted in the MOPS‐rich medium with a preliminary concentration in the range of ≈0–10 000 µg mL^−1^. The serially diluted antibiotic solutions in the MOPS rich (Teknova, #M2105) or MOPS minimal media (Teknova, #M2106) supplemented with 0.04% glucose were dispensed into the microwells. At the same time, a fresh medium without antibiotics was added to the positive control wells. Then, they were sealed with an anaerobic film (Thermo Fisher, UK) and incubated under anaerobic conditions at 37 °C for 24 h.^[^ ^4^ ^]^ All the MIC tests were performed in biological triplicate. Once all the replicates were obtained in experiments, a Hill curve was fit to the three average data as a function of the antibiotic, and the half‐maximal concentration was taken as the MBEC_50_. The lowest concentration at which no increase in OD600 was observed was considered the true MIC. All MBECs were also quantified in a similar fashion.

### Live/Dead Staining of Microcolonies

After the mature bacterial microcolonies grown in the MA hydrogels were treated with antibiotics for 24 h, they were washed with PBS three times to remove non‐adherent bacteria. The viability of microcolonies was assessed based on a commercial film tracer kit (LIVE/DEAD biofilm viability kit, Thermo Fisher), which labeled the dead and live microcolonies with the membrane potential sensitive dye propidium iodide (PI) and the nucleic acid stain SYTO‐9, respectively.^[^ ^67^ ^]^ The concentrations of PI and SYTO‐9 were 40 and 6.7 µM, respectively, in the current experiments.

### Transcriptome Sequencing of Microcolonies by Illumina HiSeq

To perform the transcriptome analysis, the bacterial microcolonies in soft and stiff MA hydrogels were first lysed with 100 µL alginate lyase (Sigma Aldrich) at the concentration of 1U mL^−1^ for 15 min at room temperature. The total RNA of each sample was extracted using Trizol reagent (Qiagen) and their concentrations were quantified by NanoDrop (Thermo Fisher Scientific Inc.). Subsequently, 1 µg of the total RNA was taken for the following library preparation. The bead‐purified double‐stranded cDNA was then treated with End Prep Enzyme Mix to repair both ends and add dA‐tailing in one reaction, followed by T‐A ligation to add adaptors at both ends. The adaptor‐ligated DNA was then size‐selected using beads to recover fragments of ≈400 bp (insert size: ≈300 bp). Each sample was amplified by PCR using P5 and P7 primers, both of which carried sequences that could anneal to the flow cell for bridge PCR.

The P5/P7 primers carried indices that allowed for multiplexing. In the experiment, the PCR products were cleaned up with beads, validated with a Qsep100 (Bioptic), and quantified with a Qubit 3.0 fluorometer (Invitrogen). Afterward, the libraries with different indices were multiplexed and loaded on an Illumina HiSeq instrument according to the manufacturer's instructions (Illumina). The sequencing was implemented using a 2 × 150 paired‐end (PE) configuration while the corresponding image analysis and base calling were conducted by the HiSeq control software (HCS) + OLB + GAPipeline‐1.6 (Illumina) on the HiSeq instrument. To remove technical sequences, the pass filter data in Fastq format were processed by Cutadapt (version 1.9.1). As such, the reference genome sequences and gene model annotation files for related species were downloaded from NCBI for mapping. Finally, the clean data were aligned with the genomic reference by the software Bowtie2 (v2.2.6). The gene expression analysis was carried out from pairwise ends by HTSeq (v0.6.1p1) in the current experiments.

### Quantification of Intracellular ATP Levels

The intracellular ATP level in *E. coli* microcolonies was determined using the enhanced ATP assay kit (Beyotime). *E. coli* (ATCC 25922) grown at 37 °C with shaking at 200 rpm were washed and resuspended to obtain an OD 600 of 0.5 with 0.01 mol L^−1^ of PBS (pH 7.4). Subsequently, the suspension of *E. coli* at the density of 1 × 10^4^ CFU mL^−1^ was mixed into the MA pregel solution to form the bacterium‐embedding MA hydrogel samples with specific stiffness. The samples were then cultured in the MOPS‐rich medium at 37 °C for 48 h. All the bacterial cultures were centrifuged at 12 000 rpm at 4 °C for 5 min and washed with PBS twice times. The bacterial precipitates were lysed by lysozyme and ATP detection lysate and centrifugated at 12 000 rpm at 4 °C for 5 min. The resulting supernatant was used for intracellular ATP characterization. Specifically, 100 µL of detection solution was added to each well of a standard 96‐well plate and incubated at room temperature for 5 min. Afterward, the supernatants were mixed into the wells and the corresponding luminescence intensities were recorded immediately with a Varioscan^TM^ LUX multimode microplate reader (Thermo Fisher).

### ROS Measurement

The levels of ROS in the bacterial microcolonies grown in soft and stiff MA hydrogels were detected by a reactive oxygen species assay kit (Beyotime). In brief, the MA pregel solution mixed with the suspension of *E. coli* at the density of 1 × 10^4^ CFU mL^−1^ was crosslinked with UV light of 365 nm to fabricate the hydrogel samples with specific stiffness, as described in the above‐mentioned section. Subsequently, the samples were cultured in the MOPS‐rich medium in a 37 °C incubator for 48 h. 2′,7′‐dichlorofluorescein diacetate (DCFH‐DA) with a final concentration of 10 µmol L^−1^ was required to co‐incubated with the bacterial microcolonies at 37 °C for 30 min in each well. After they were washed with 0.01 mol L^−1^ of PBS three times, the intensities of fluorescein isothiocyanate (FITC) with the emission wavelength of 525 nm were immediately quantified using a Varioscan^TM^ LUX multimode microplate reader (Thermo Fisher).

### Mouse Thigh Infection Models

In the mouse thigh infection model, 0.1 mL of *E. coli* suspension (at a dose of 1.0 × 10^8^ CFU per mouse) was injected into the right thigh of BALB/c female mice (*n* = 6 per group). The mice were treated 1 day after infection with a specified intraperitoneal administration of PBS, fumarate (10 mg kg^−1^), colistin (8 mg kg^−1^), or the combination of fumarate with colistin (i.e., 5 + 0.5, 10 + 4, and 10 + 8 mg kg^−1^). The mice were euthanized by cervical dislocation 72 h after infection. After incubation at 37 °C for 24 h, the right thighs were aseptically removed, homogenized, serially diluted, and plated on TSA to count bacteria. To assess the toxicity of this treatment combination, these thigh specimens and different organs including heart, liver, spleen, lung, and kidney were removed from each group (*n* = 3) mice for histological analysis by hematoxylin and eosin staining. The care and operation of animals followed the international standards on animal welfare. The animal protocols were approved by the Institutional Animal Care and Use Committee of Yi Shengyuan Gene Technology (Tianjin) Co., Ltd. (protocol number YSY‐DWLL‐2022093).

## Conflict of Interest

The authors declare no conflict of interest.

## Supporting information

Supporting information

## Data Availability

The data that support the findings of this study are available from the corresponding author upon reasonable request.
