# Supplementary material for: Extracellular Matrix Rigidities Regulate the Tricarboxylic Acid Cycle and Antibiotic Resistance of Three‐Dimensionally Confined Bacterial Microcolonies
Source: Adv Sci (Weinh). 2023 Jan 19;10(9):2206153. doi: 10.1002/advs.202206153 (PMC10037996; doi:10.1002/advs.202206153)
Supplement: Supplementary file 1 — Supporting information [file ADVS-10-2206153-s001.pdf]

Supporting Information

Extracellular matrix rigidities regulate the tricarboxylic acid cycle and antibiotic resistance of three-dimensionally confined bacterial microcolonies

Yiming Han[1,2], Nan Jiang[1,2], Hongwei Xu[1,2], Zuoying Yuan[1,2], Jidong Xiu[1,2], Sheng Mao[1,2], Xiaozhi Liu[3], and Jianyong Huang[1,2,]*

[1]*Department of Mechanics and Engineering Science, College of Engineering, Peking University, Beijing 100871, China*

[2]*Beijing Innovation Center for Engineering Science and Advanced Technology, College of Engineering, Peking University, Beijing 100871, China*

[3]*Tianjin Key Laboratory of Epigenetics for Organ Development of Premature Infants, Fifth Central Hospital of Tianjin, Tianjin, 300450, China*

*Correspondence should be addressed to J. Huang (jyhuang@pku.edu.cn).

Table of Contents:

Figures S1~S6

Table S1

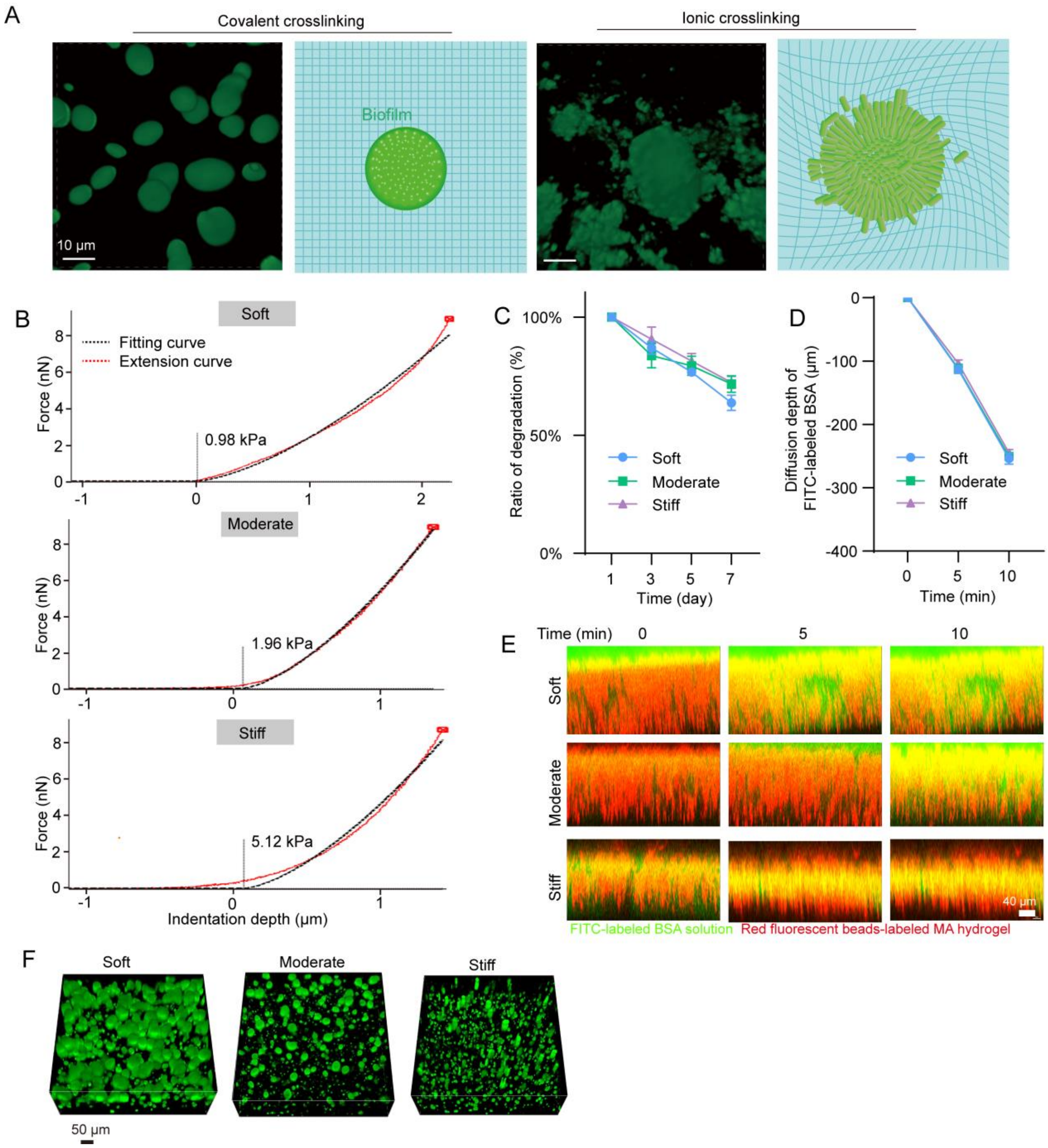

Figure S1. (A) Representative confocal images of bacterial microcolonies grown in the MA hydrogels, which were covalently crosslinked wih Irgacure 2959 (photoinitiator) with the aid of UV light at the wavelength of 365 nm, and ionically crosslinked with the Ca^{2+} solutions, respectively. Scale bar = 50 μm.

(B) Representative curves of applied forces versus indentation depths presented by the atomic force microscope (AFM) tests for soft, moderate, and stiff MA hydrogels that were covalently crosslinked.

(C) Statistical results on degradation rates of soft, moderate, and stiff MA hydrogels which were covalently crosslinked with Irgacure 2959 with the help of UV light.

(D) Time-dependent diffusion of FITC-labelled bovine serum albumin (BSA) solutions in the soft, moderate, and stiff MA hydrogels.
(E) representative images describing the spatiotemporal dynamics of FITC-labelled BSA in the hydrogel samples, where the FITC-labelled BSA proteins and the hydrogel networks were denoted as green and red, respectively. Note that, the hydrogel samples were randomly embedded with red fluorescence beads of 200 nm for visualization of the hydrogel samples based on confocal imaging.
(F) 3D confocal images of *E. coli* microcolonies grown in the soft, moderate, and stiff MA hydrogels. Scale bar =50 μm.

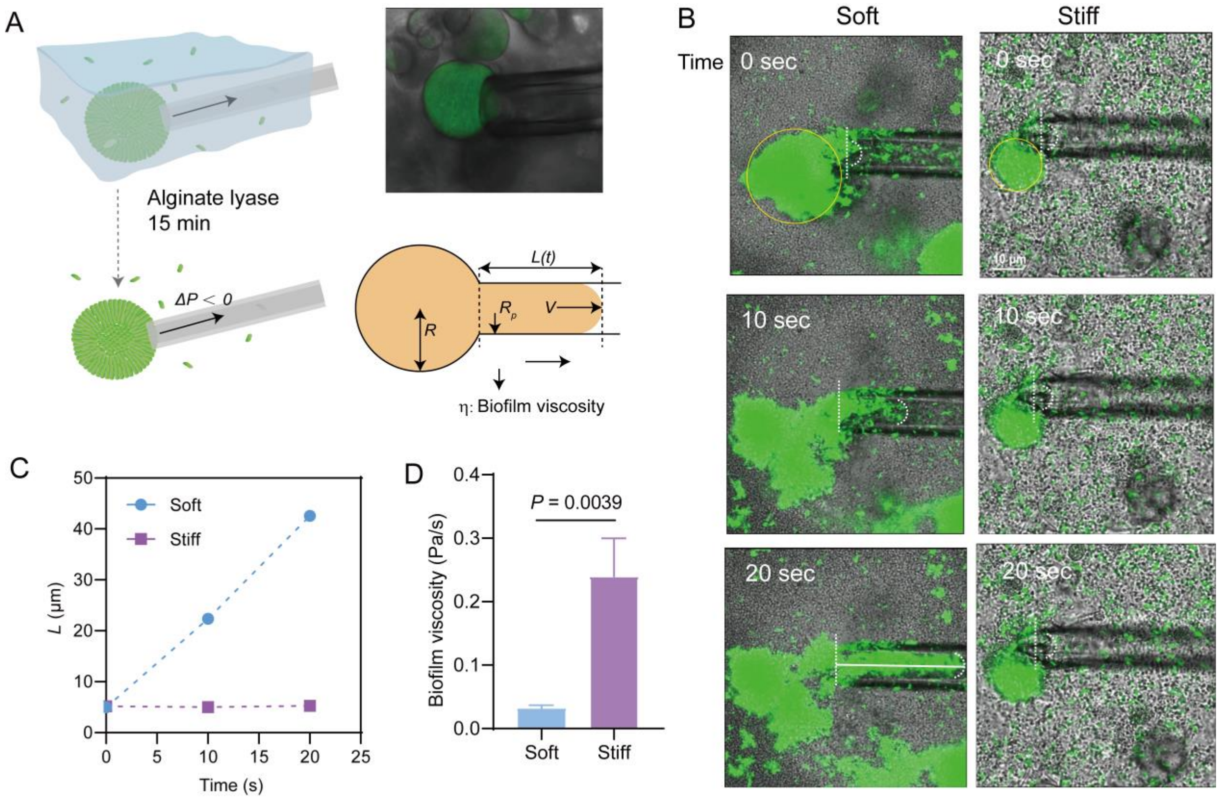

Figure S2. Mechanical characterization of *E. coli* microcolonies using micropipette aspiration.

(A) The process of capturing a bacterial microcolony with a micropipette (diameters: 7~15 μm). The bottom-right image presented the geometric parameters in the process of capturing a bacterial microcolony (*R*, aggregate radius; *Rp*, pipette radius; *L(t)*, tongue length at instant t; *DP*, applied negative pressure; v, tongue progression speed in the pipette; η , microcolony viscosity)

(C) Bright-field temporal evolution and (D) statistic progression of bacterial microcolonies embedded in the soft and stiff matrix inside the pipette (tongue length, *L*) at 0, 10, 20 secs after the application of 100 Pa negative pressures. Curves were representative of n = 3 independent experiments.

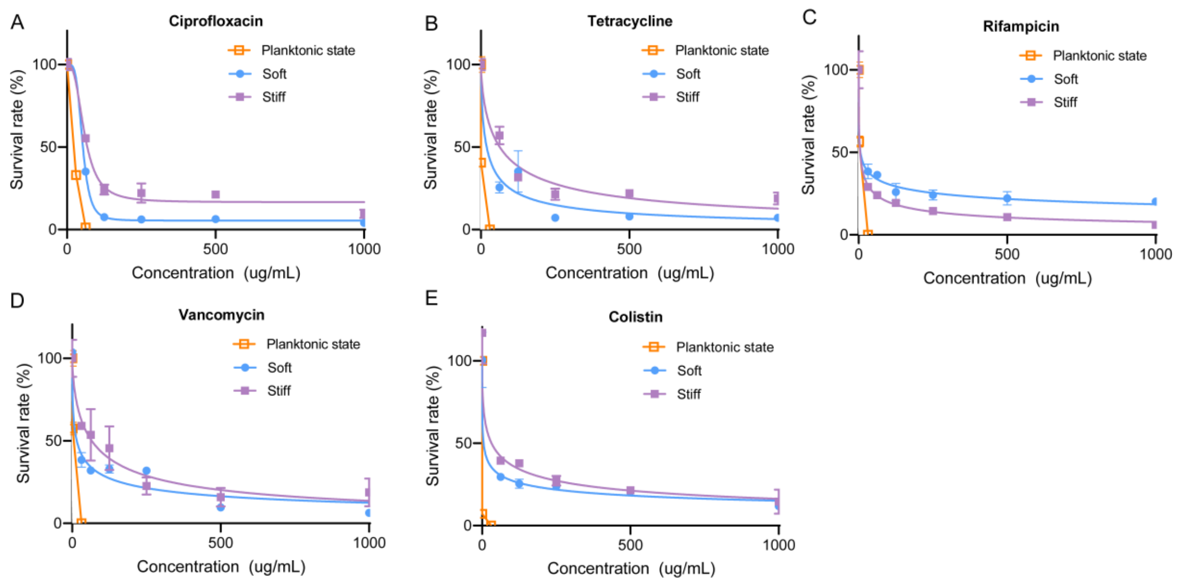

Figure S3. MIC curves of single planktonic *E. coli* and MEBC curves of *E. Coli* microcolonies cultured in soft and stiff MA hydrogels, which were treated with ciprofloxacin (A), tetracycline (B), rifampicin (C), vancomycin (D), and colistin (E), respectively, in the rich media.

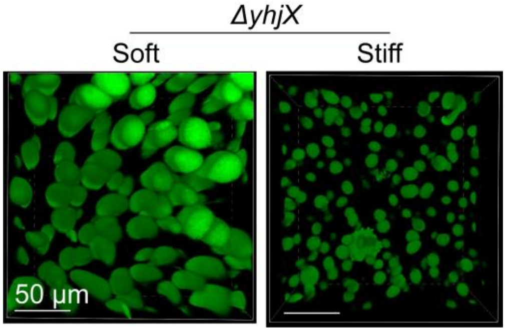

Figure S4. Representative 3D confocal images of *ΔyhjX* microcolonies in the soft and stiff hydrogels. Scale bar = 50μm.

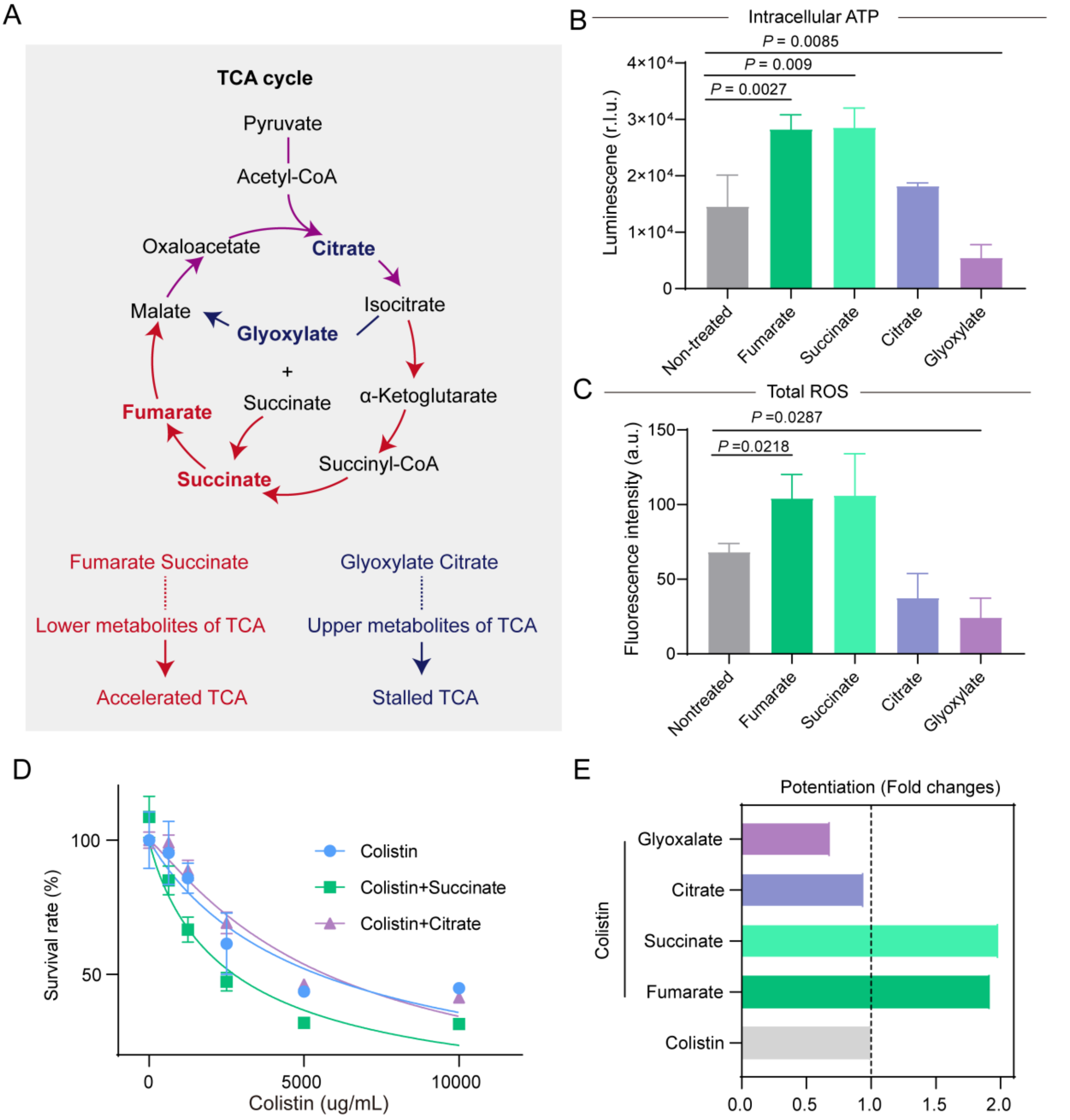

Figure S5. (A) Process of the TCA cycle and critical intermediate metabolites.
(B) Quantification of intracellular ATP and (C) total ROS of the mature microcolonies in the stiff hydrogel samples, which were treated for 4 h with the minimal medium (0.04% glucose) containing serial dilutions of colistin in the presence of 15 mM fumarate, 24 mM succinate, 22.4 mM citrate, and 30 mM glyoxylate, respectively.
(D) Curves of survival rates of mature bacterial microcolonies in 3D MA hydrogels regulated by succinate and citrate. In these experiments, the microcolonies were treated for 4 h with the minimal medium (0.04% glucose) containing serial dilutions of colistin in the presence of succinate (24 mM) or citrate (22.4 mM). All data were shown as Mean ± SD (n = 6).
(E) Colistin in combination with fumarate, succinate, citrate, and glyoxylate enhanced antibiotic susceptibility of mature bacterial colonies in 3D matrices. Note that potentiation

was defined as the fold change in $MBEC_{50}$ of the combination treatment relative to that of the colistin treatment alone.

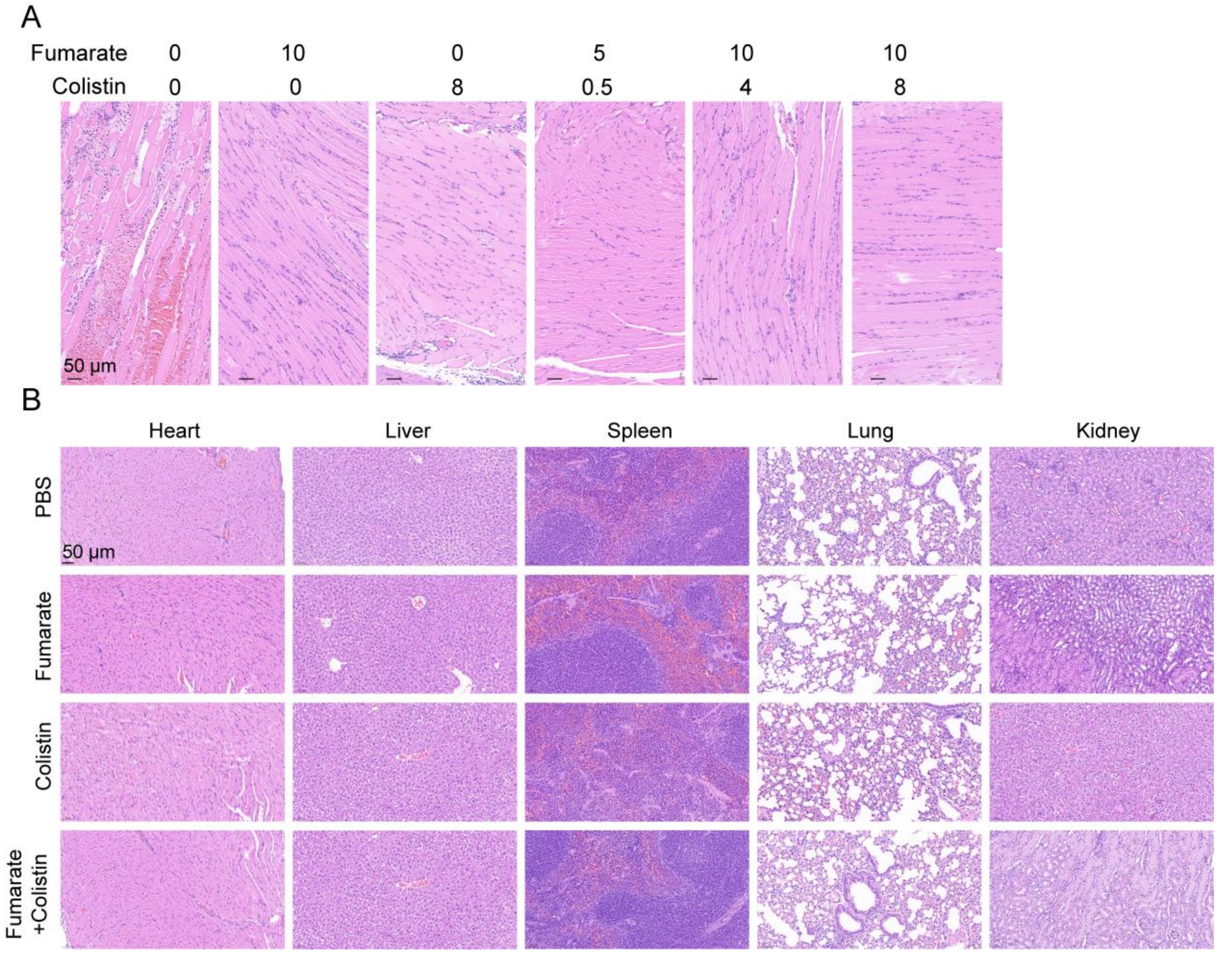

Figure S6. Histopathological features of thighs (A) and different organs (B) in the mouse peritonitis-sepsis model and the thigh infection model treated with PBS (control), fumarate (10 mg kg^{-1}), colistin (8 mg kg^{-1}), or their combination (10 mg kg^{-1} fumarate + 8 mg kg^{-1} colistin). The results were representative of three biological replicates.

Table S1. Preparation conditions of covalently crosslinked MA hydrogels with different rigidities.

Samples	2% MA (μL)	Concentration of irgacure(*w/v*)	Curing time (sec)	Young's modulus (kPa) (Mean± SD)
Soft	100	0.075%	60	0.85±0.12
Moderate	100	0.1%	120	1.96±0.22
Stiff	100	0.1%	300	5.12±1.50
